# Supplementary material for: piR-39980 mediates doxorubicin resistance in fibrosarcoma by regulating drug accumulation and DNA repair
Source: Commun Biol. 2021 Nov 19;4:1312. doi: 10.1038/s42003-021-02844-1 (PMC8605029; doi:10.1038/s42003-021-02844-1)
Supplement: Supplementary file 2 — Supplementary Information [file 42003_2021_2844_MOESM2_ESM.pdf]

SUPPLEMENTARY INFORMATION

piR-39980 mediates doxorubicin resistance in fibrosarcoma by regulating drug accumulation and DNA repair

Basudeb Das[1], Neha Jain[1], and Bibekanand Mallick[1,*]

[1]RNAi and Functional Genomics Lab., Department of Life Science, National Institute of Technology Rourkela, Rourkela-769008, Odisha, India

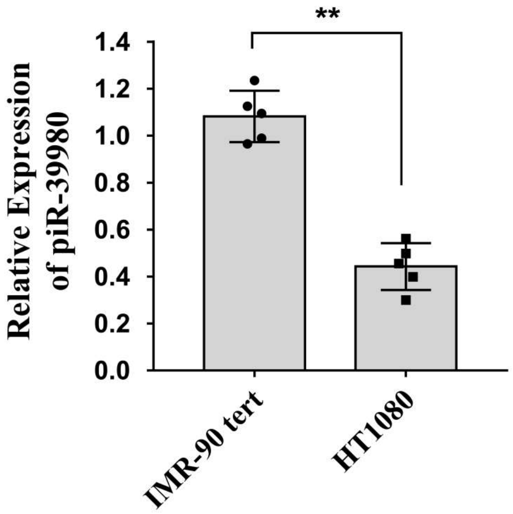

Supplementary Figure 1: Expression of piR-39980 in HT1080 cells compared to IMR90 cells. Bars, mean ± SEM; $n = 5$ independent experiments; $^{**}P < 0.01$, t test.

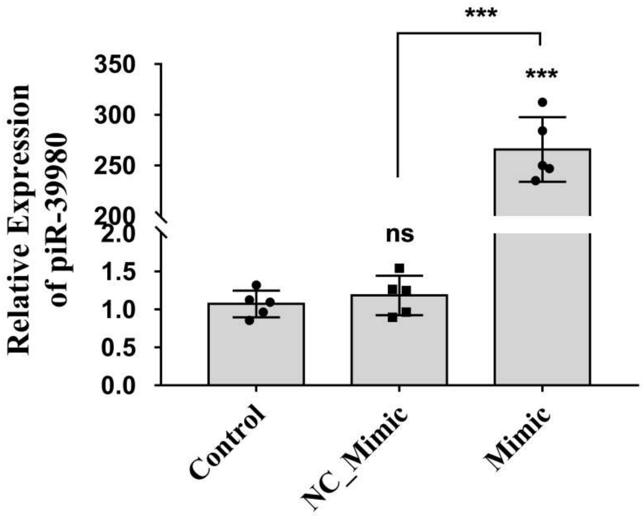

Supplementary Figure 2: Relative expression of piR-39980 in HT1080 cells transfected with 20 nM piR-39980 mimics compared with untransfected control and NC_Mimic. Bars, mean ± SEM; $n = 5$ independent experiments; ns = non-significant, $^{***}P < 0.001$, Tukey's multiple comparisons test.

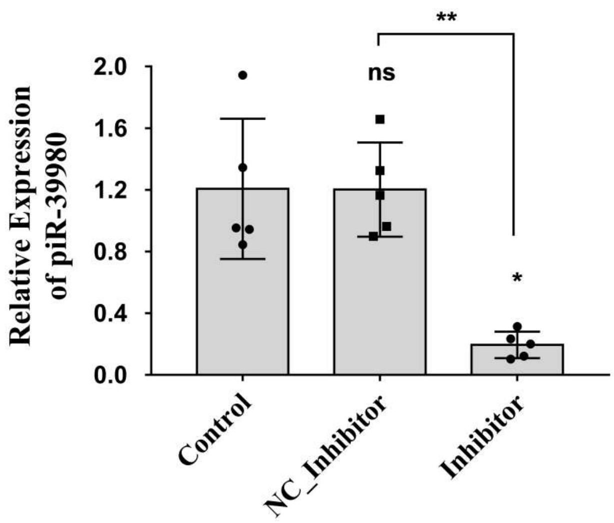

Supplementary Figure 3: Relative expression of piR-39980 in HT1080 cells transfected with 20 nM piR-39980 inhibitors compared with untransfected control and NC_Inhibitor. Bars, mean ± SEM; *n* = 5 independent experiments; ns = non-significant, **P* < 0.05, ***P* < 0.01, Tukey's multiple comparisons test.

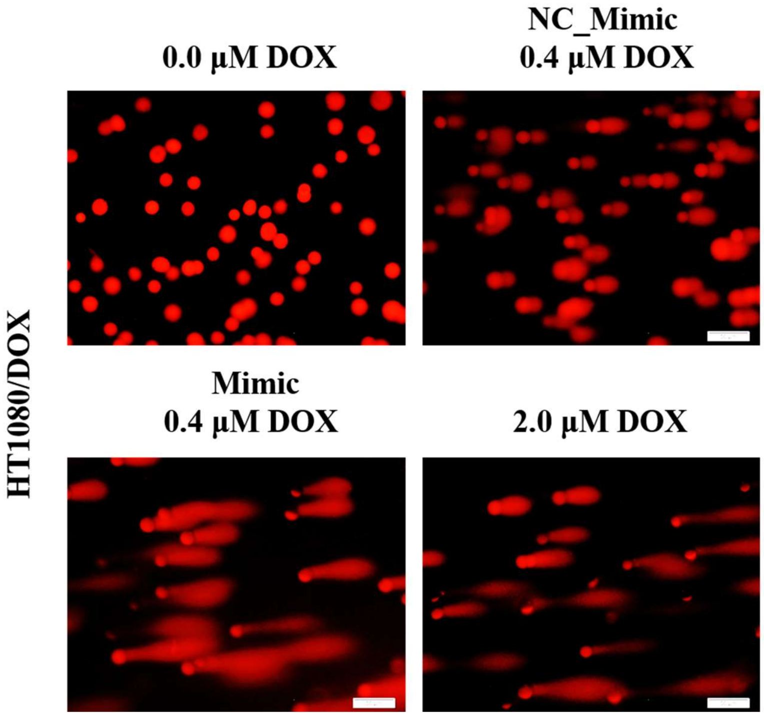

Supplementary Figure 4: Effect of piR-39980 on DOX-induced DNA damage determined by comet assay.

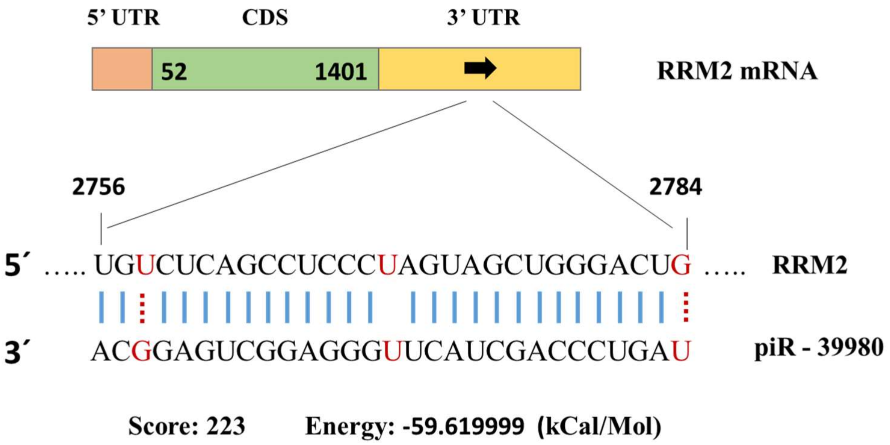

Supplementary Figure 5: The target binding site of piR-39980 within 3′UTR of RRM2

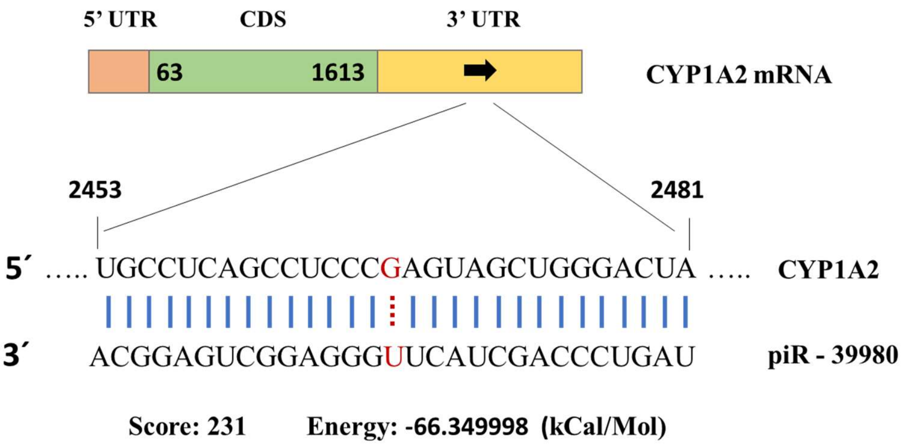

Supplementary Figure 6: The target binding site of piR-39980 within 3′UTR of CYP1A2

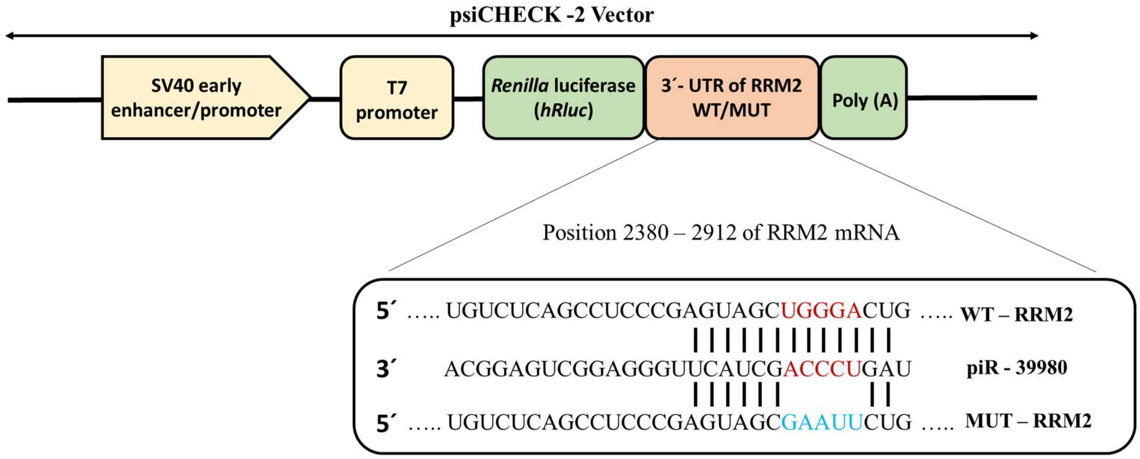

Supplementary Figure 7: Dual-luciferase reporter assay construct of RRM2

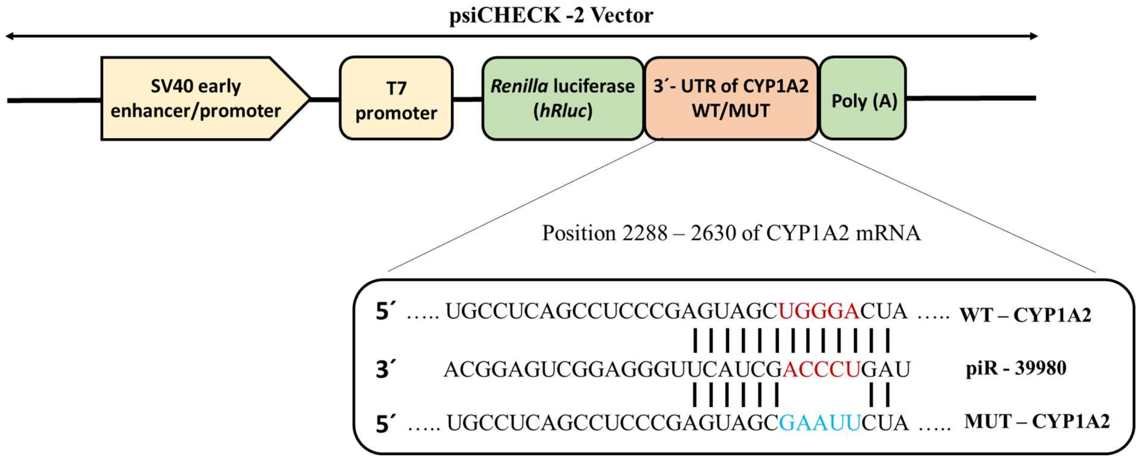

Supplementary Figure 8: Dual-luciferase reporter assay construct of CYP1A2

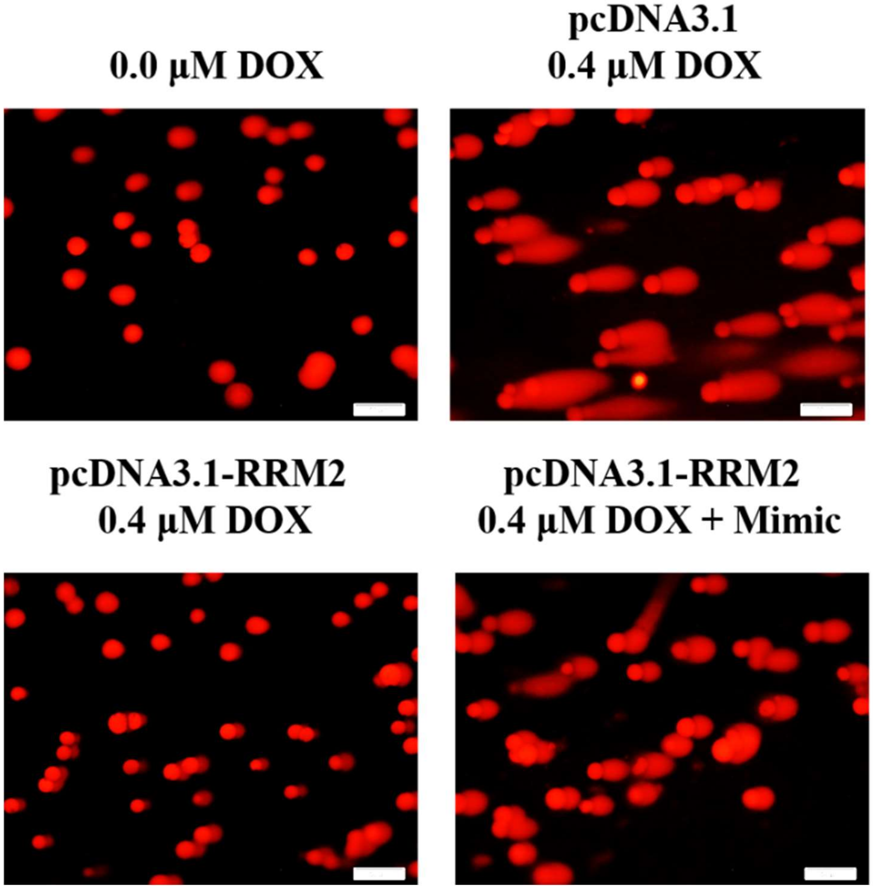

Supplementary Figure 9: Effect of piR-39980 on DOX-induced DNA damage determined by comet assay.

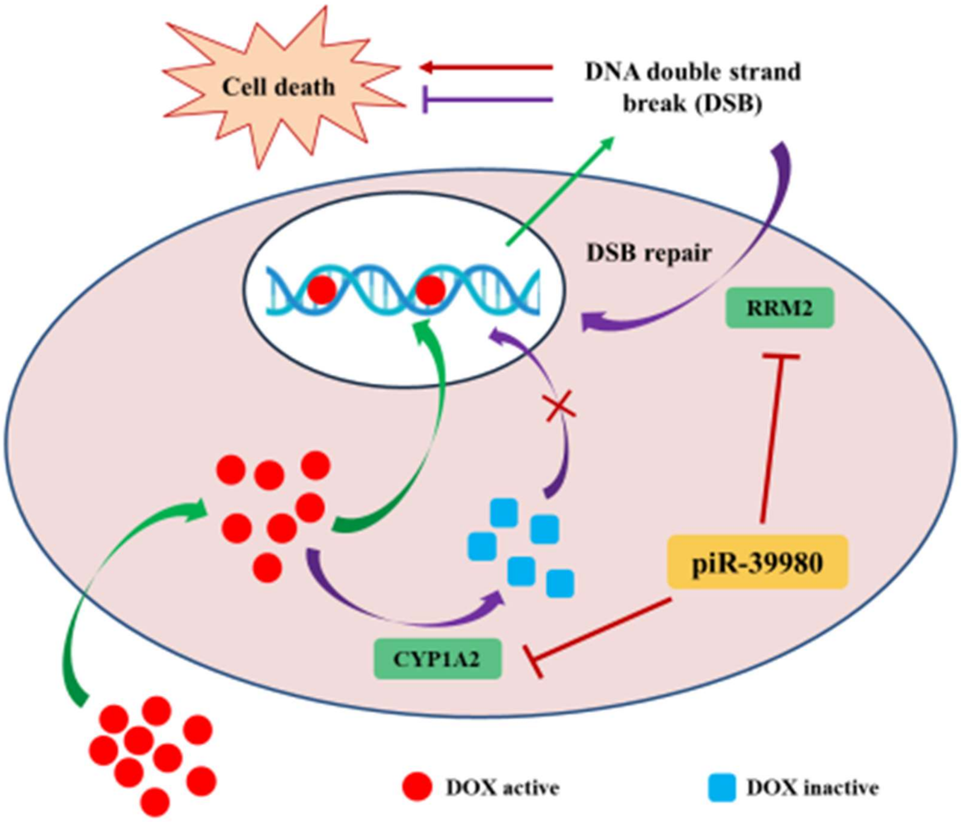

Supplementary Figure 10: A model showing molecular mechanisms of piR-39980 inducing DOX sensitivity of fibrosarcoma cells through its targets, RRM2 and CYP1A2.

In general, the drug-metabolizing enzyme CYP1A2 inactivates DOX within cells, whereas RRM2 induces a repair mechanism and rescues DOX-mediated DNA damage, both of which confers resistance. However, piR-39980 boosts cell death by increasing DOX sensitivity by repressing these two genes, RRM2 and CYP1A2, two targets of this piRNA.

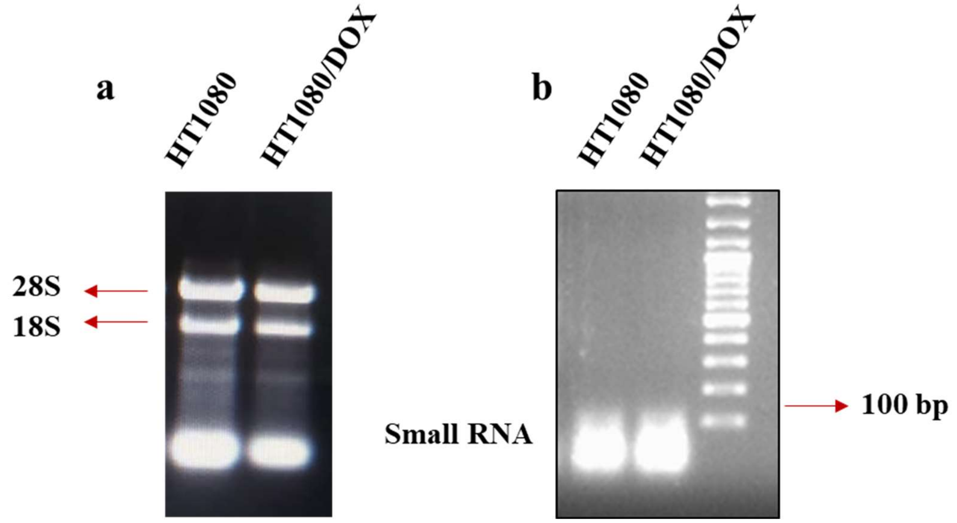

Supplementary Figure 11: Agarose gel electrophoresis showing the integrity of RNA isolated from the parental HT1080 and HT1080/DOX cells. a Total RNA was isolated using HiPurA™ Total RNA Miniprep Purification Kit (Himedia). **b** Small RNA was isolated using *mir*Vana™ miRNA Isolation Kit (Invitrogen).

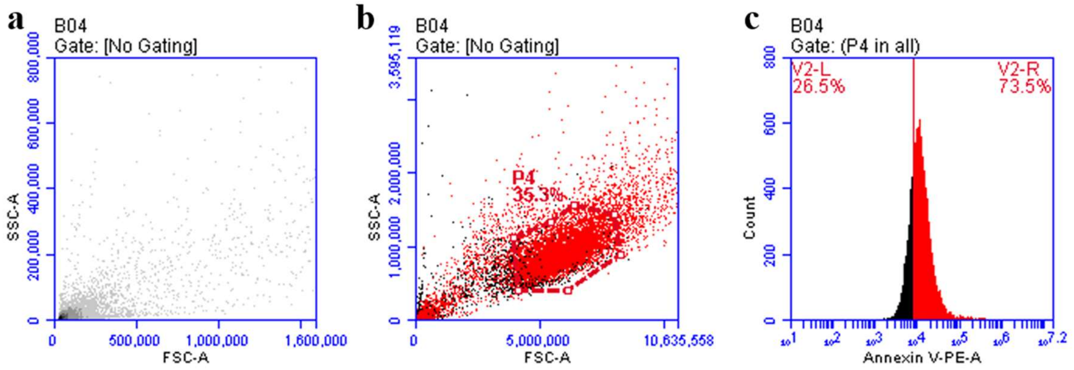

Supplementary Figure 12: Flow cytometry gating strategy. Representative image of Fig. 5c. Data were analyzed using BD Accuri^{TM} C6 Plus flow cytometry. First, we created a dot plot of the data displaying SSC vs FSC. We placed a gate around the cell in the dense area. The debris was visible in the lower-left corner of the plot, which was excluded by the gate. FSC/SSC gate showed 20-40% cell populations that vary from sample to sample. Then, we created a histogram and plotted the gated data. The histogram showed cell count vs annexin V-PE stain. We made a boundary at 10^4 on X-axis. The left quadrant shows unstained cells, and the right quadrant shows annexin V-PE-stained cells.

Supplementary Table 1: Sequences of piR-39980 Mimic, Inhibitor, and NC_Inhibitor

**piR-39980 mimic (5´-3´):**<br>rUrArGrUrCrCrCrArGrCrUrArCrUrUrGrGrGrArGrGrCrUrGrArGrGrCmA
**piR-39980 inhibitor (5´-3´):**<br>mC/ZEN/mAmGmCmCmUmCmCmCmAmAmGmUmAmGmCmUmGmGmGmAmCmUmA/3ZEN/
**NC_Inhibitor (5´-3´):**<br>mG/ZEN/mCmGmAmCmUmAmUmAmCmGmCmGmCmAmAmUmAmUmGmG/3ZEN/

Supplementary Table 2: List of primers and their sequences used in this study

piRNA/Genes (GenBank ID)		Primer sequence (5´-3´)
piR-39980 (DQ601914.1)	F	TAGTCCCAGCTACTTGGGAGG
	R	Universal reverse primer (Provided by Qiagen with kit)
U6 (NR_004394.1)	F	CTCGCTTCGGCAGCACATATACT
	R	ACGCTTCACGAATTTGCGTGTC
RRM2_qRT-PCR (NM_001165931.1)	F	ACTATGCTCTCCCTCCGTGT
	R	CGGTCCAAAAGGAAGCCTCT
CYP1A2_qRT-PCR (NM_000761.5)	F	CTGGGCACTTCGACCCTTAC
	R	TCTCATCGCTACTCTCAGGGA
RRM2_luciferase (NM_001165931.1)	F	GCGGCACTCGAGACTTTAGTAGGAAACCATGAGC
	R	ATAGCGGCCGCTGTCTCAGCTTTCTTCTCCC
CYP1A2_luciferase (NM_000761.5)	F	GACGCTCGAGAAGTGTCGAATGACTTCTAGTGT
	R	ATAGCGGCCGCGGTGGTTCATACCTGTTAATC
RRM2_overexpression (NM_001165931.1)	F	ACTCTCGAGATGGGAAGGGTCGGAGGCA
	R	GTCGGATCCCCTGATTCCAACTTCCGCC
CYP1A2_overexpression (NM_000761.5)	F	ACGCTCGAGATGGCATTGTCCCAGTCTGTTC
	R	GTCGGATCCCACTGCACTCCAGCTTAGGAG
RPL13 (NM_000977.4)	F	GTTCGGTACCACACGAAGGT
	R	TACGGAGACTAGCGAAGGCT
