# Supplementary material for: piR-39980 mediates doxorubicin resistance in fibrosarcoma by regulating drug accumulation and DNA repair
Source: Commun Biol. 2021 Nov 19;4:1312. doi: 10.1038/s42003-021-02844-1 (PMC8605029; doi:10.1038/s42003-021-02844-1)
Supplement: Supplementary file 3 — Description of Additional Supplementary Files [file 42003_2021_2844_MOESM3_ESM.pdf]

Description of Additional Supplementary Files

File name: Supplementary Data 1
Description: All source data underlying the graphs and charts.
