# Supplementary material for: piR-39980 mediates doxorubicin resistance in fibrosarcoma by regulating drug accumulation and DNA repair
Source: Commun Biol. 2021 Nov 19;4:1312. doi: 10.1038/s42003-021-02844-1 (PMC8605029; doi:10.1038/s42003-021-02844-1)
Supplement: Supplementary file 5 — Reporting Summary [file 42003_2021_2844_MOESM5_ESM.pdf]

nature research

Corresponding author(s): Bibekanand Mallick COMMSBIO-21-1419B

Last updated by author(s): Oct 24, 2021

Reporting Summary

Nature Research wishes to improve the reproducibility of the work that we publish. This form provides structure for consistency and transparency in reporting. For further information on Nature Research policies, see our Editorial Policies and the Editorial Policy Checklist.

Statistics

For all statistical analyses, confirm that the following items are present in the figure legend, table legend, main text, or Methods section.

n/a	Confirmed	
☐	☒	The exact sample size (n) for each experimental group/condition, given as a discrete number and unit of measurement
☐	☒	A statement on whether measurements were taken from distinct samples or whether the same sample was measured repeatedly
☐	☒	The statistical test(s) used AND whether they are one- or two-sided<br>*Only common tests should be described solely by name; describe more complex techniques in the Methods section.*
☒	☐	A description of all covariates tested
☐	☒	A description of any assumptions or corrections, such as tests of normality and adjustment for multiple comparisons
☐	☒	A full description of the statistical parameters including central tendency (e.g. means) or other basic estimates (e.g. regression coefficient) AND variation (e.g. standard deviation) or associated estimates of uncertainty (e.g. confidence intervals)
☐	☒	For null hypothesis testing, the test statistic (e.g. F, t, r) with confidence intervals, effect sizes, degrees of freedom and P value noted<br>*Give P values as exact values whenever suitable.*
☒	☐	For Bayesian analysis, information on the choice of priors and Markov chain Monte Carlo settings
☒	☐	For hierarchical and complex designs, identification of the appropriate level for tests and full reporting of outcomes
☒	☐	Estimates of effect sizes (e.g. Cohen's d, Pearson's r), indicating how they were calculated

Our web collection on statistics for biologists contains articles on many of the points above.

Software and code

Policy information about availability of computer code

Data collection	GenBank, NCBI
Data analysis	BD AccuriTM C6, OpenComet v1.3, ImageJ, miRanda, GraphPad Prism 7.0.

For manuscripts utilizing custom algorithms or software that are central to the research but not yet described in published literature, software must be made available to editors and reviewers. We strongly encourage code deposition in a community repository (e.g. GitHub). See the Nature Research guidelines for submitting code & software for further information.

Data

Policy information about availability of data

All manuscripts must include a data availability statement. This statement should provide the following information, where applicable:

- Accession codes, unique identifiers, or web links for publicly available datasets
- A list of figures that have associated raw data
- A description of any restrictions on data availability

All study data are included in the article and/or supporting information.

Field-specific reporting

Life sciences study design

All studies must disclose on these points even when the disclosure is negative.

Sample size	*Describe how sample size was determined, detailing any statistical methods used to predetermine sample size OR if no sample-size calculation was performed, describe how sample sizes were chosen and provide a rationale for why these sample sizes are sufficient.*
Data exclusions	*Describe any data exclusions. If no data were excluded from the analyses, state so OR if data were excluded, describe the exclusions and the rationale behind them, indicating whether exclusion criteria were pre-established.*
Replication	All the experiments were performed three independent times in triplicates. All the data in this study are reproducible.
Randomization	*Describe how samples/organisms/participants were allocated into experimental groups. If allocation was not random, describe how covariates were controlled OR if this is not relevant to your study, explain why.*
Blinding	*Describe whether the investigators were blinded to group allocation during data collection and/or analysis. If blinding was not possible, describe why OR explain why blinding was not relevant to your study.*

Reporting for specific materials, systems and methods

We require information from authors about some types of materials, experimental systems and methods used in many studies. Here, indicate whether each material, system or method listed is relevant to your study. If you are not sure if a list item applies to your research, read the appropriate section before selecting a response.

Materials & experimental systems

n/a	Involved in the study	
☐	☒	Antibodies
☐	☒	Eukaryotic cell lines
☒	☐	Palaeontology and archaeology
☒	☐	Animals and other organisms
☒	☐	Human research participants
☒	☐	Clinical data
☒	☐	Dual use research of concern

Methods

n/a	Involved in the study	
☒	☐	ChIP-seq
☐	☒	Flow cytometry
☒	☐	MRI-based neuroimaging

Antibodies

Antibodies used	Recombinant Anti-gamma H2A.X (phospho S139) antibody [EP854(2)Y]; Abcam, Cambridge, UK; ab81299; Rabbit monoclonal [EP854(2)Y].<br>Goat Anti-Rabbit IgG H&L (DyLight® 488) preadsorbed; Abcam; ab96899;
Validation	ab81299 has been referenced in 89 publications.<br>Yu B et al. Mitochondrial phosphatase PGAM5 modulates cellular senescence by regulating mitochondrial dynamics. Nat Commun 11:2549 (2020)<br>Sousounis K et al. Eya2 promotes cell cycle progression by regulating DNA damage response during vertebrate limb regeneration. Elife 9:N/A (2020).<br>Zhang X et al. Vitamin C Protects Porcine Oocytes From Microcystin-LR Toxicity During Maturation. Front Cell Dev Biol 8:582715 (2020).<br>Novak J et al. Interleukin-1a associates with the tumor suppressor p53 following DNA damage. Sci Rep 10:6995 (2020).

Eukaryotic cell lines

Policy information about cell lines

Cell line source(s)	Human fibrosarcoma cell line (HT1080) and Human embryonic kidney cell line 293 (HEK293) were used in the study.<br>Both the cell lines were obtained from the cell repository of the National Centre for Cell Science (NCCS), Pune, Maharashtra, India.
Authentication	Short Tandem Repeat (STR) analysis
Mycoplasma contamination	Cell lines were tested negative from Mycoplasma contamination.
Commonly misidentified lines<br>(See ICLAC register)	*Name any commonly misidentified cell lines used in the study and provide a rationale for their use.*

Flow Cytometry

Plots

Confirm that:

- [x] The axis labels state the marker and fluorochrome used (e.g. CD4-FITC).
- [x] The axis scales are clearly visible. Include numbers along axes only for bottom left plot of group (a 'group' is an analysis of identical markers).
- [ ] All plots are contour plots with outliers or pseudocolor plots.
- [x] A numerical value for number of cells or percentage (with statistics) is provided.

Methodology

Sample preparation	To determine whether piR-39980 boosts DOX-mediated apoptosis, we performed flow cytometry using PE Annexin V Apoptosis Detection Kit I (BD, Franklin Lakes, NJ, USA; 559763). HT1080 cells were seeded in a 6-well tissue culture plate (Tarsons; 980010) at 2 x 105 cells/well and cultured for 24 h. Then, the cells were transfected with 20 nM mimic/inhibitor and corresponding negative controls. After 24 h of transfection, cells were treated with 0.4 µM DOX and incubated for 48 h. Then, the cells were harvested with 0.25% trypsin (Himedia; TCL007), washed twice with ice-cold 1X PBS, and resuspended in 500 ml binding buffer. 5 ml Annexin V-PE was added with the samples and incubated for 15–20 min in the dark. Cells were then analyzed using BD AccuriTM C6 Plus flow cytometry (BD, USA) within 1 h.<br>Human fibrosarcoma cell line (HT1080) was obtained from the cell repository of the National Centre for Cell Science (NCCS), Pune, Maharashtra,
Instrument	BD AccuriTM C6 Plus flow cytometry (BD, USA)
Software	BD AccuriTM C6
Cell population abundance	A single cell line is used in this study. Cells are not contaminated with other cells which is authenticated by STR analysis
Gating strategy	Gating strategy was used to gate out cellular debris. First, we created a dot plot of the data displaying SSC vs FSC. We placed a gate around the cell. The debris were visible in the lower left corner of the plot which was excluded by the gate. FSC/SSC gate showed 20-40% cell populations that varies from sample to sample. Then, we created a histogram and plot the gated data. The histogram showed cell count vs annexin V-PE stain. We made a boundary at 10^4 on X-axis. Left quadrant shows unstained cells and right quadrant shows annexin V-PE stained cells. We used unstained cell sample for all the experiments to defined the boundary between unstained cell and stained cells. These details including a figure exemplifying the gating strategy is included in the Supplementary Information file.

- [x] Tick this box to confirm that a figure exemplifying the gating strategy is provided in the Supplementary Information.
